# Predictors of atypical language lateralization in focal epilepsy: A mega‐analysis of fMRI evidence

**DOI:** 10.1111/epi.18422

**Published:** 2025-05-05

**Authors:** Freya Prentice, Lara Chehabeddine, Maria Helena Eriksson, Jennifer Murphy, Leigh N. Sepeta, William D. Gaillard, Madison M. Berl, Frédérique Liégeois, Torsten Baldeweg

**Affiliations:** ^1^ Developmental Neurosciences Research and Teaching Department UCL Great Ormond Street Institute of Child Health London UK; ^2^ Department of Neuropsychology Great Ormond Street Hospital for Children London UK; ^3^ Clinical Research Institute, Department of Internal Medicine American University of Beirut Beirut Lebanon; ^4^ Department of Epidemiology and Population Health American University of Beirut Beirut Lebanon; ^5^ Program in Neuroscience and Mental Health The Hospital for Sick Children Research Institute Toronto Ontario Canada; ^6^ School of Psychology University of Surrey Guildford UK; ^7^ Center for Neuroscience Research, Children's National Hospital George Washington University Washington District of Columbia USA; ^8^ Division of Neuropsychology Children's National Hospital Washington District of Columbia USA; ^9^ Department of Psychiatry and Behavioral Sciences George Washington University Washington District of Columbia USA

**Keywords:** epilepsy surgery, fMRI, language lateralization, language plasticity

## Abstract

**Objective:**

To identify predictors of language lateralization derived from functional magnetic resonance imaging (fMRI) in children and adults with left‐ and right‐sided focal epilepsy.

**Methods:**

We conducted a mega‐analysis of data from 914 individuals from 24 samples. We used multilevel models to identify predictors of language lateralization in left and right hemisphere epilepsy groups. We assigned each participant a clinical predictor score to explore whether there was a cumulative influence of predictors on increasing atypical language lateralization.

**Results:**

Left hemisphere epilepsy was a predictor of greater atypical language lateralization in the combined sample. Additional predictors of atypical language lateralization included left/ambidextrous handedness in both the left and right hemisphere groups, and longer duration of epilepsy, frontal lobe involvement, and history of a stroke or other precipitating injury in the left hemisphere group only. There was a cumulative effect of predictors in the left hemisphere groups. Eighty percent of individuals with four or more predictors had atypical language lateralization, compared to 19% of individuals with no predictors, other than left hemisphere epilepsy.

**Significance:**

Consistent with theories of language plasticity, we demonstrated a robust effect of early acquired left hemisphere injury on language lateralization. There was also a subtle effect of duration of epilepsy, perhaps reflecting increasing bilaterality with age in adulthood. The association between left/ambidextrous handedness and atypical language lateralization in the left and right hemisphere groups likely reflects both genetic and epilepsy‐associated effects. The total number of predictors identified for an individual could serve as an indication for presurgical language fMRI, when surgical management is considered.

**Key points**

- In our mega‐analysis of 914 participants, we identified robust predictors of atypical language lateralization.
- Atypical language lateralization was associated with epilepsy in the left hemisphere; however, it was still common in the right hemisphere epilepsy groups.
- There was a robust effect of early left hemisphere acquired injury on atypical language lateralization, presumably reflecting the effects of early plasticity.
- A longer duration of epilepsy was associated with atypical language lateralization, perhaps akin to the effect of health aging on increasing bilaterality.
- The effect of predictors was cumulative, whereby the total number of predictors serves as an indicator of atypical language lateralization in individual patients.

## 1 INTRODUCTION

Children and adults with focal epilepsy show higher rates of right and bilateral patterns of language lateralization than those seen in the general population (25%–30% in focal epilepsy vs 5% in healthy controls),^1^ as identified using task‐based functional magnetic resonance imaging (fMRI). This higher prevalence of atypical language lateralization in individuals with focal epilepsy is presumed to reflect either the disruption of the typical trajectory of left language specialization^2^, ^3^ or interhemispheric reorganization of language networks from the left hemisphere (LH) to the right hemisphere (RH).^4^, ^5^, ^6^ Accordingly, atypical language lateralization has been associated with a seizure focus or epileptogenic lesion in the LH.^7^, ^8^, ^9^, ^10^, ^11^, ^12^

There is converging evidence that atypical language lateralization is more common in individuals with an early as opposed to late onset of seizures.^6^, ^13^, ^14^, ^15^ This is also consistent with theories of language plasticity that predict that atypical language lateralization is more likely when an insult is sustained at an early age due to less specialization in language networks and greater potential for reorganization early in development.^3^, ^5^, ^16^, ^17^ In a recent meta‐analysis of more than 1000 individuals, we found a small but significant correlation between earlier age at seizure onset and atypical language lateralization (Prentice et al., preprint^18^). Other studies have shown reorganization after early acquired insults predating the onset of seizures including stroke and other precipitating injuries.^19^, ^20^, ^21^ These findings all suggest a critical role of early plasticity in language lateralization in focal epilepsy.

Other potential predictors of language lateralization in epilepsy, distinct from the effects of early plasticity, include duration of epilepsy,^22^ left and ambidextrous handedness,^6^, ^20^, ^23^ a mesial temporal lobe focus^24^ including hippocampal sclerosis (HS),^25^ structural asymmetries^26^, ^27^ and interictal epileptiform discharges.^9^ However, only a few studies have examined how such variables interact to affect language lateralization, and these studies have been limited to temporal lobe epilepsy samples^23^ or studies with small sample sizes (*n* < 50).^22^ A study on a small sample of adults with left HS identified predictors of atypical language lateralization (determined by Wada test) and demonstrated that a greater number of predictors was predictive of atypical language lateralization, suggesting that there may be a cumulative effect of those variables on language lateralization.^21^

There is a need to identify predictors of language lateralization in a large and heterogenous focal epilepsy cohort. A 2017 study found that 50% of European epilepsy centers using fMRI for language lateralization recommended patients for presurgical fMRI if atypical language lateralization was suspected.^28^ Given that clinical indicators of atypical language lateralization are currently used in the selection of candidates for presurgical fMRI, identification of predictors of language lateralization could improve selection of candidates. In addition, some individuals are unable to complete a successful fMRI, and so in these cases, prediction of language lateralization from clinical variables is especially pertinent.

Here we conducted a mega‐analysis of 914 individuals with focal epilepsy to identify predictors of language lateralization and examine the cumulative influence of such predictors.

## 2 MATERIALS AND METHODS

### 2.1 Mega‐analysis dataset

The multicenter dataset used here was created as part of a previous meta‐analysis examining the association between age at seizure onset and language lateralization.^18^ We carried out a literature search on August 14 2024, and we screened articles that included (1) individual with epilepsy and (2) fMRI for language lateralization. Screening resulted in the inclusion of 33 articles plus two additional pediatric datasets. Where data were available in the article or supplement, we extracted fMRI laterality indices (LIs) and clinical characteristics. Where data were not available, we requested them from the authors. Information on the side of epilepsy, age at seizure onset, and LIs were available for 1157 individuals across 35 samples. For this study specifically, we excluded samples with missing information on the localization of epilepsy, handedness, duration of epilepsy, and etiology. This resulted in a final sample of 914 individuals from 24 samples, BA including 646 with LH epilepsy and 268 with RH epilepsy. More detail on the methods for the search, screening, and data extraction can be found in the preprint.^18^ A table containing the characteristics of each sample included in this study can be found in the Data S1.

### 2.2 Measures

#### 2.2.1 Laterality indices

We used continuous LIs calculated from task‐based fMRI as a measure of language lateralization. LIs range from +1 to −1, indicating left to right language lateralization. LIs from the different samples were calculated using different language tasks and region of interest (ROI). Language tasks used for the LIs selected included verbal fluency (phonemic and semantic fluency, verb generation), auditory description, semantic decision, and a conjunction of multiple tasks. For those studies that reported LIs for multiple tasks, we chose tasks that robustly activated frontal regions, such as verbal fluency. Similarly, where studies reported LIs for multiple ROIs, we chose frontal ROIs, which have been shown to be reproducible^29^ and robust lateralizing^30^ in healthy adults. The language and baseline task and ROI used for each sample can be found in Table S1. In a sub‐analysis, we looked at predictors of language lateralization using LIs calculated in temporal ROI only, using receptive language tasks expected to produce appropriate temporal lobe activation (i.e., excluding fluency tasks). For descriptive statistics, LIs were categorized into right (−1 to −.2), bilateral (−.2 to .2), and left language representation (.2 to +1).

#### 2.2.2 Predictor variables

All included participants had information on age at seizure onset, duration of epilepsy, side of epilepsy, location of epilepsy, pathology of epilepsy, handedness, and a history of insults predating the onset of seizures. We coded individuals as having LH or RH epilepsy and excluded those with no side of epilepsy reported or those with bilateral epilepsy. We coded whether the structural abnormality involved the frontal or temporal lobes. We coded scans that were reported as “MRI negative” as not having any known lobar location, irrespective of seizure focus.

Handedness was determined in several different ways across samples: using the Edinburgh Handedness Inventory or by clinical evaluation or self‐report. The method used by each sample can be seen in Table S1. Individuals were coded as being right‐handed vs left‐handed or ambidextrous. We categorized insults predating seizure onset into two separate groups: stroke and other precipitating injury, which included febrile seizures, infection, and head trauma.

### 2.3 Analysis strategy

#### 2.3.1 Identification of predictors of language lateralization

Due to the negative skew in the residuals of these models we performed an exponential transformation of the LI values to reduce the skew. All statistical analyses used transformed LI values while the data visualization used untransformed LI values. As the data came from different samples we used a multilevel modeling approach, with the sample included as a random effect. Given that the different samples vary based on the language and baseline tasks used, ROI and LI calculation method, as well as the characteristics of the sample (see Table S1), “sample” best modeled the overall methodological variation. As fixed effects we included variables that have been previously associated with language lateralization: age at seizure onset, duration of epilepsy, handedness, HS diagnosis, stroke, other precipitating injuries, and the involvement of frontal or temporal regions, given their role in language functions.

In LH and RH samples separately, we entered all variables into a multilevel model, including sample as a random effect. We explored potential multicollinearity in the model by examining the Variance Inflation Factor (VIF). If the VIF was higher than 2 for one or more variables, we removed the variable with the highest VIF and reran the model. We repeated this procedure until all variables had a VIF under 2. We compared the Akaike information criteria (AIC) and Bayes factor (BF) of the final models with a model with random effects only, and ran chi‐square tests, to choose the model with best fit. In addition, we examined the interactions between side of epilepsy and all other variables in the combined sample, to determine whether predictors had a significantly different effect on language lateralization in the LH and RH epilepsy groups (see Data S1). We reran these analyses in a subset of the sample who also had temporal LIs (LH = 514, RH = 167).

#### 2.3.2 Calculation of a “predictor score” for language lateralization

Variables that were found to be significant independent predictors in the final multilevel models were used to derive a cumulative “predictor score” for each individual in the LH and RH group. For example, if only a history of stroke and HS diagnosis were identified as significant predictors, and an individual had a history of stroke but no HS, they would receive a predictor score of 1. We used one‐way analyses of variance (ANOVAs) to compare the effect of “number of predictors” (i.e., an individual's predictor score) on language lateralization to examine whether there was a cumulative effect of predictors on increasingly right lateralized language. To examine significance with each additional predictor, we used post hoc pairwise comparison and applied the Benjamini–Hochberg correction.

#### 2.3.3 Identification of predictors of left/ambidextrous handedness

Given that handedness has been previously identified as a predictor of language lateralization in both focal epilepsy^6^, ^20^, ^23^ and in the general population,^31^, ^32^ and that rates of left handedness are greater in focal epilepsy populations,^33^ we also examined which epilepsy characteristics were associated with handedness beyond language lateralization. We used multilevel logistic regression models to examine the interaction between side of epilepsy and other included variables on handedness and applied the Benjamini–Hochberg correction to control for multiple comparison. The results of these analyses are presented in the Data S1.

## 3 RESULTS

### 3.1 Sample characteristics

The sample included 646 individuals with LH epilepsy and 268 with RH epilepsy. There were similar number of children (55%) and adults, and the median age at fMRI scan was 16.6 years (interquartile range [IQR] = 12.5–32.4). Most individuals had an onset of epilepsy in childhood (before 18 years; 82%), with a median age at seizure onset of 8.3 years (IQR = 4–14). Language tasks used for LI calculation included fluency (58%), auditory decision (22%), semantic decision (9%), or a conjunction of language tasks (6%), with all other tasks making up less than 5% of the sample. LI were calculated primarily using frontal ROI (80%), although some used a more extensive frontotemporal (17%) or whole brain ROI (3%). Handedness was determined primarily through clinical evaluation (51%), the Edinburgh Handedness Inventory or other standardized measures (28%), or self‐report (1%). For the other 20% of patients, the method of handedness determination was not specified. Descriptive statistics for the demographic and clinical characteristics of the LH and RH epilepsy samples can be found in Table 1.

**TABLE 1:** Demographic and clinical characteristics of the LH and RH epilepsy samples.

	LH epilepsy sample	RH epilepsy samples
Age at fMRI scan, median [IQR]	17 years [12.7, 34]	16.1 years [11.8, 29.2]
Female sex, %	55%	56%
Age at seizure onset, median [IQR]	8 years [4, 14]	9 years [4, 13]
Duration of seizures, median [IQR]	7.95 years [3.9, 16]	7.58 years [3.95, 14]
Left/ambidextrous handedness, %	22%	16%
Frontal involvement, %	22%	20%
Temporal involvement, %	74%	74%
Hippocampal sclerosis diagnosis, %	36%	33%
Stroke, %	5%	1%
Other precipitating injury, %	17.5%	16%

Abbreviations: fMRI, functional magnetic resonance imaging; IQR, interquartile range; LH, left hemisphere; RH, right hemisphere.

### 3.2 Side of epilepsy

In the LH epilepsy sample, 34% of individuals had atypical language lateralization (20% right lateralized, 14% bilateral) and the median LI was .49 (IQR = −.08 to .73). In the RH epilepsy sample, 23% of individuals had atypical language lateralization (12% right lateralized, 11% bilateral) and the median LI was .61 (IQR = .25 to .80). In the combined LH and RH sample, LH epilepsy was a significant predictor of greater atypical language lateralization (β = −.22, *p* < .001).

### 3.3 Identification of predictors of language lateralization

For the LH multilevel model, the fixed effects explained 19% of the variance in LI, and the random effect explained an additional 3% (AIC = 1227). There was very strong evidence (BF > 1000) that this model was a better fit for the data than one with random effects only (AIC = 1302), and the difference was statistically significant (*p* < .001). Multicollinearity was low (VIF below 2). For the RH multilevel model, the fixed effects explained 7% of the variance in LI, and the random effects explained an additional 15% (AIC = 538). There was strong evidence (BF > 1000) that this model was a poorer fit for the data than one with random effects only (AIC = 510), and there was no statistically significant difference between the models (*p* > .05). Multicollinearity was low (VIF below 2). The estimates of the fixed effects for both the LH and RH epilepsy models are displayed in Table 2.

**TABLE 2:** Estimates of fixed effects for LH and RH epilepsy sample multilevel models.

Model	Fixed effects	Estimate	t	*p* value
LH epilepsy	Age at seizure onset	−.00	−.20	.843
	Duration of epilepsy	−.01	−3.50	< .001***
	Left/ambidextrous handedness	−.39	−6.39	< .001***
	Frontal lobe involvement	−.16	−2.34	.019**
	Temporal lobe involvement	.03	.39	.694
	Hippocampal sclerosis diagnosis	.05	.72	.471
	Stroke	−.45	−3.86	< .001***
	Other precipitating injury	−.28	−4.14	< .001***
RH epilepsy	Age at seizure onset	.01	1.76	.081
	Duration of epilepsy	.00	.24	.814
	Left/ambidextrous handedness	−.36	−3.55	< .001***
	Frontal lobe involvement	.09	.88	.380
	Temporal lobe involvement	−.02	−.22	.825
	Hippocampal sclerosis diagnosis	.04	.36	.723
	Stroke	−.35	−1.01	.316
	Other precipitating injury	.04	.34	.731

Abbreviations: LH, left hemisphere; RH, right hemisphere.

* *p* < .05.

** *p* < .01.

*** *p* < .001.

**FIGURE 1:**
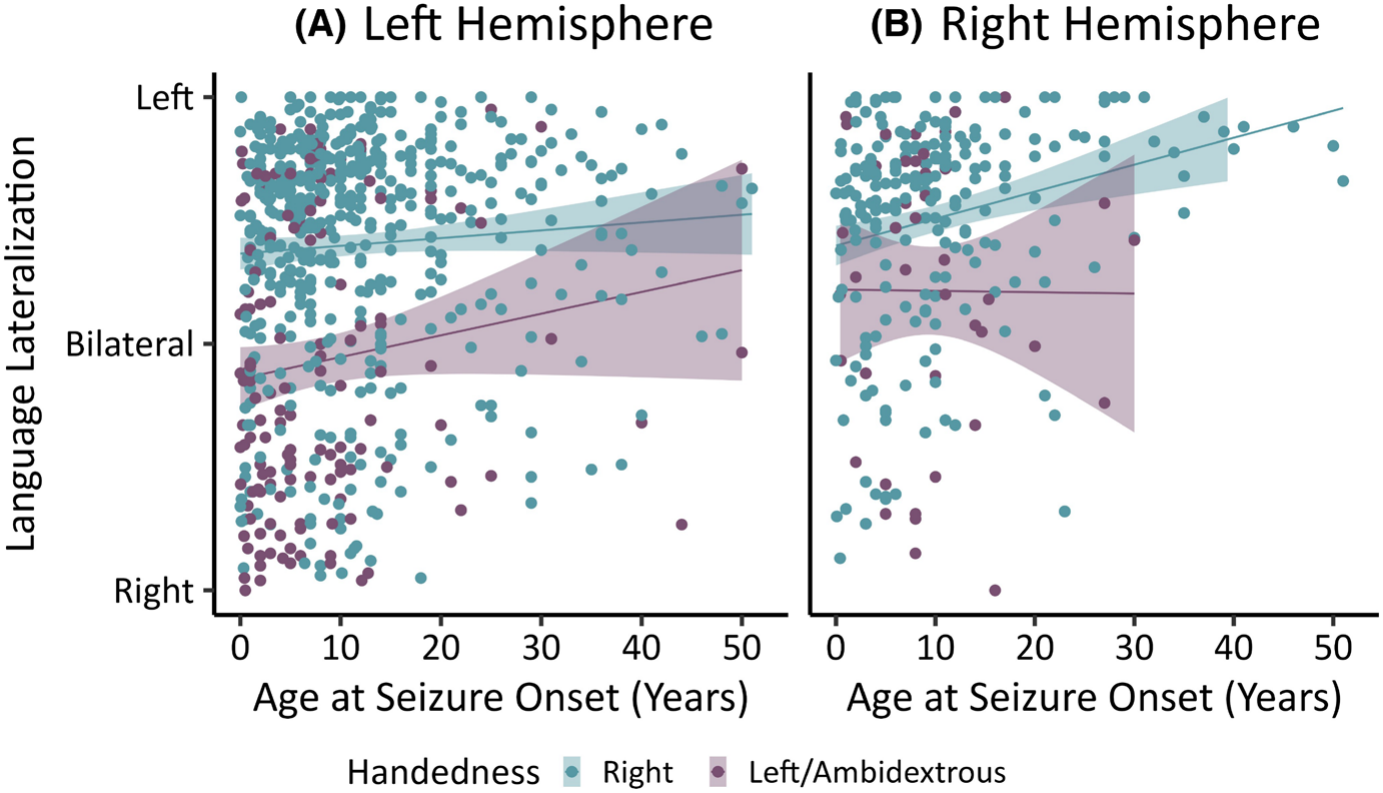
Distribution of language lateralization (laterality indices) and age at seizure onset in left/ambidextrous (yellow) and right‐handed individuals (turquoise) in the (A) left and right (B) hemisphere epilepsy groups.

Left/ambidextrous handedness was the only variable that was significantly predictive of atypical language lateralization in the final LH and RH models. Figure 1 shows the correlation between age at seizure onset and language lateralization in right vs left/ambidextrous handed individuals in the LH and RH epilepsy groups.

In the subset of patients with LIs calculated in temporal ROI, left/ambidextrous handedness (β = −.20, *p* = .032) and stroke (β = −.35, *p* = .034) were the only significant predictors of temporal language lateralization in the LH epilepsy group, and there were no significant predictors in the RH epilepsy group. The full results of this analysis can be found in the Data S1.

### 3.4 Interactions with side of epilepsy

**FIGURE 2:**
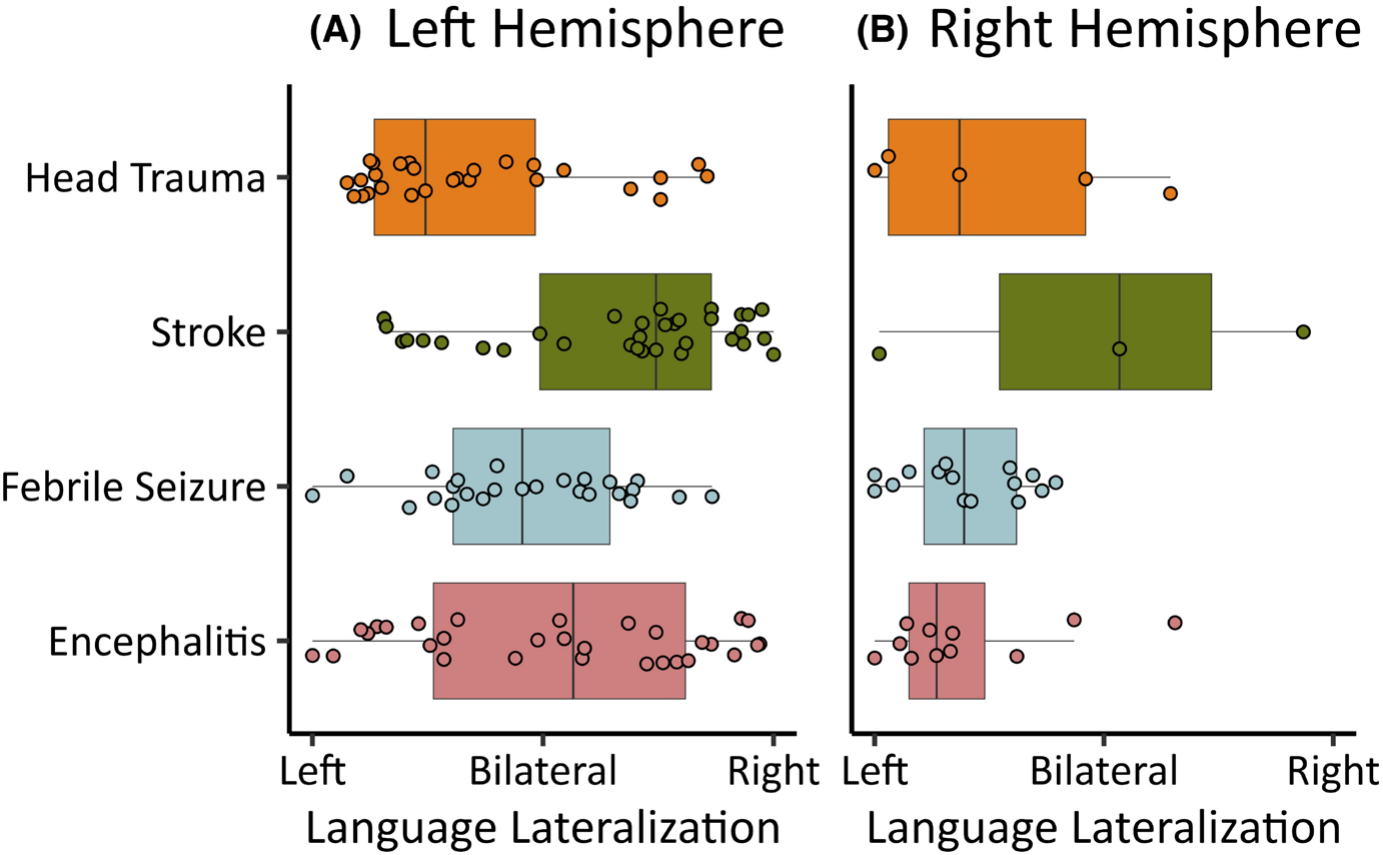
Language lateralization (laterality indices) in individuals with an insult predating seizure onset in the left (A) and right (B) hemisphere epilepsy groups.

In the combined sample, there were significant interactions of LH epilepsy, with a longer duration of epilepsy (β = −.01, *p* = .026), frontal involvement (β = −.29, *p* = .030), and a history of precipitating injuries (β = −.44, *p* = .003) on language lateralization. These variables were associated with atypical language lateralization in the LH but not RH epilepsy groups. The interaction effects between side of epilepsy and all other variables can be found in the Data S1. Figure 2 shows the language lateralization patterns for those with LH and RH epilepsy, with the most common injuries reported to predate seizure onset: stroke, head trauma, febrile seizures, and encephalitis.

### 3.5 Cumulative influence of predictor variables on language lateralization

**FIGURE 3:**
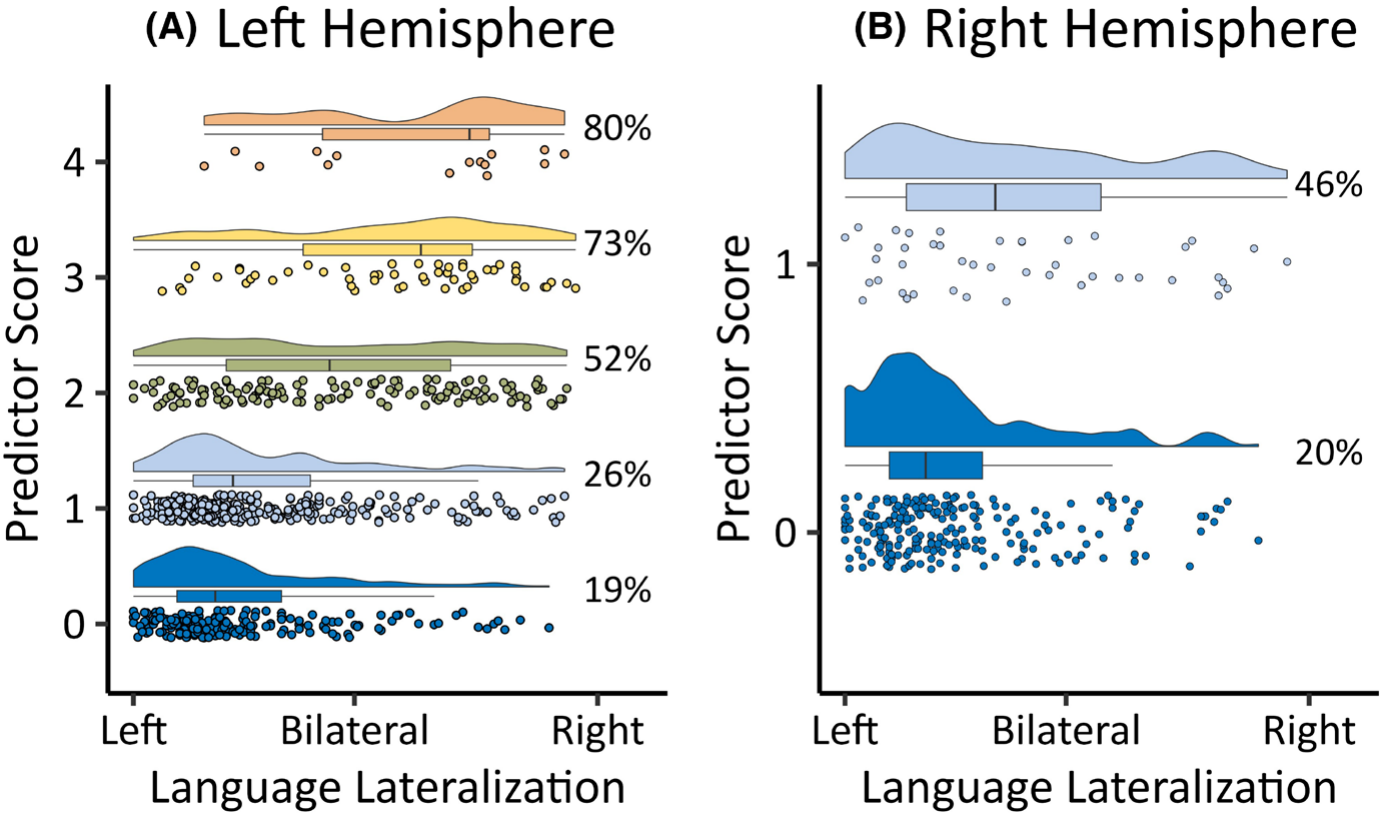
Cumulative influence of the number of predictors on atypical language lateralization (laterality indices) in the left (A) and right (B) hemisphere samples and the percentage of individuals in each predictor score group with atypical language lateralization.

In the LH sample, individuals could have up to five predictors (longer duration of epilepsy, left/ambidextrous handedness, frontal lobe involvement, stroke, or other precipitating injuries), although no individuals had all five predictors. Duration of epilepsy was coded categorically as “short” vs “long” using median duration for the total sample (≥7.8 years), given the lack of theoretical or empirical evidence for a cutoff above which duration of epilepsy has a greater effect. Eighty percent of those with four predictors were atypically lateralized, compared to 19% of those with zero predictors. Atypical language lateralization increased with the number of predictors (*F* (4, 641) = 32.92, *p* < .001). Post hoc comparisons showed that there was a difference in LI between groups with zero vs one predictor (*p* = .007), one vs two predictors (*p* < .001), and two vs three predictors (*p* = .008), but not three vs four predictors (*p* = .557) (see Figure 3A). In the RH sample, individuals could have one predictor (left/ambidextrous handedness); 46% of those with this predictor were atypically lateralized, compared to 20% of those with no predictors. There was an effect of the number of predictors on LI (*F* (1, 266) = 13, p < .001) (Figure 3B). When limiting the LH and RH sample to those with no predictors, LH epilepsy was no longer a significant predictor of atypical language lateralization in a multilevel model (β = −.004, *p* = .914).

## 4 DISCUSSION

Our study identified predictors of fMRI language lateralization in a large focal epilepsy sample of 914 individuals, including both LH and RH epilepsy groups.

### 4.1 Acquired insults drive language reorganization

We found that a history of insults predating the onset of habitual seizures (including stroke) was an independent and robust predictor of greater atypical language lateralization in the LH group. It is possible that these early precipitating injuries may partially drive the previously demonstrated association between younger age at seizure onset and atypical language lateralization,^6^, ^13^, ^14^, ^15^, ^18^ as seen with the inclusion of stroke patients in a previous study.^20^ Furthermore, the inclusion of these variables may explain why age at seizure onset was not a predictor of language lateralization in the final model for the LH group. Given that early theories of language plasticity were primarily based on children with early diffuse and hemispheric insults,^4^, ^5^ it is perhaps unsurprising that early acquired LH injury was a more robust predictor of reorganization than the age at seizure onset alone.

Our findings indicate that an acquired LH insult, such as a stroke or encephalitis, was associated with atypical language lateralization. It is possible that the higher rates of atypical language lateralisaiton in this group are due to both the sudden onset of acquired insults and more extensive damage to eloquent cortex compared to more focal and slow‐growing pathologies. Consistent with this, not all precipitating injuries had the same magnitude of effect on language lateralization, and in our multilevel model, stroke was the strongest predictor (Figure 2). As expected, early injury in the LH has a significantly greater effect on language lateralization than early RH injury. LH stroke was also a predictor of atypical language lateralization in the temporal lobe, suggesting that this type of early acquired injury is associated with extensive reorganization across frontal and temporal language regions. Overall, these findings demonstrate that acquired LH insults predating the onset of habitual seizures may be a key driver of language reorganization and serve, clinically, as an indicator for right or bilateral language representation.

### 4.2 Chronicity effect of seizures on language lateralization

Looking beyond the strong effect of an acquired injury on language reorganization, there may also be a more subtle influence of ongoing seizure activity. In the LH group, a longer duration of epilepsy was associated with increasing atypical language lateralization (as previously shown in a small pediatric sample^22^). This may differ in nature from the more extensive reorganization associated with early LH injury, and instead reflect increasing functional adaptation or compensation from the RH. This is akin to the influence of healthy aging on increasing bilateral language representation^14^, ^31^ (see the HAROLD model, Hemispheric Asymmetry Reduction in Older Adults^34^).

Future research should examine whether such increasing bilaterality with age is seen in epilepsy and disentangle the contribution of healthy aging and chronic seizures on changes in language lateralization with age. Examination of not only the difference in activation between the two hemispheres, but the changes in absolute activation in each hemisphere, as has been done in development,^3^ may help us to distinguish between reorganization and compensation mechanisms. In addition, the use of functional connectivity measures may also disentangle reorganization and compensation, both during development and aging. A functional connectivity measure has been developed that compares inter‐ and intrahemispheric connectivity of language regions during resting‐state fMRI.^35^, ^36^ This measure could have consequences for understanding the efficiency of reorganization and providing additional information for preoperative evaluation.

### 4.3 Handedness

Left and ambidextrous handedness was a predictor of greater atypical language lateralization across both LH and RH groups. This was contrary to a previous study showing that the relationship between handedness and language lateralization was specific to those with a LH epilepsy focus (in an adult temporal lobe epilepsy sample).^23^ In addition, left and ambidextrous handedness also predicted atypical language lateralization in the temporal lobe, although this was only significant in the LH epilepsy group. The presence of the association between atypical language lateralization and handedness in both the LH and RH groups, at least in the frontal lobe, may partially reflect the higher rates of atypical language lateralization in left‐handed individual in the general population.^31^, ^32^ However, there may also be an added effect of epilepsy on handedness^6^, ^13^ and the relationship between handedness and language lateralization has been shown to be stronger in individuals with LH epilepsy than in controls.^33^

Consistent with this, we demonstrated that left/ambidextrous handedness was associated with frontal involvement in the LH but not the RH epilepsy groups (see Data S1). It is possible that this reflects either joint reorganization of language and handedness in response to a left frontal lobe pathology, or a facilitative role of reorganization of one on the other. Overall, the role of handedness as a predictor of language lateralization may reflect both genetic and epilepsy‐associated effects in the LH group, but predominantly the former in the RH group. Information on family sinistrality in future studies would help to disentangle the contributions of these two factors.

### 4.4 Atypical language lateralization in individuals with RH epilepsy

In the RH group, we were unable to identify predictors of language lateralization, beyond left/ambidextrous handedness. This may be due in part to the smaller sample size and greater between‐sample variance (random‐effects) compared to the LH sample. However, atypical language lateralization was still higher in the RH epilepsy group than we would expect in the general population (20% in right handers, 46% of left handers). Figure 1 shows a cluster of patients with RH epilepsy with atypical language lateralization and an onset of epilepsy in childhood. An earlier age at seizure onset was a borderline predictor of atypical language lateralization in this group (*p* = .081). This may reflect the putative role of the right hemisphere in language development in early childhood.^3^ In addition, atypical language lateralization was demonstrated in two of the three RH stroke patients included in our sample (see Figure 2). Both of these patients remained right‐handed, suggesting that they did not have preexisting atypical language lateralisation associated with left‐handedness. The potential causes of atypical language lateralization in individuals with RH epilepsy remain uncertain.

Notably, there was no difference in rates of atypical language lateralization between LH and RH epilepsy groups with no predictors (19% and 20%, respectively). This may indicate that at least some of the effect of seizures on language lateralization is of a bilateral nature. Given that there is often a contralateral spread of seizure activity, this could contribute toward the general increase in atypical language lateralization rates in individuals with epilepsy. Atypical language lateralization has been linked to the spread of seizure activity ipsilaterally in the LH^37^ but not to the RH. A small case series of LH patients showed no association between contralateral spread of seizure activity to the RH and atypical language lateralization.^7^ However, further examination of this question in future research is needed and may help to elucidate the higher rates of atypical language lateralization in individuals with RH epilepsy compared to the general population.

### 4.5 Cumulative influence of individual predictors

Our analyses suggested that there was a cumulative effect of predictors on language lateralization, whereby having a greater number of predictors was associated with increasing atypical language lateralization. Given that suspected atypical language lateralization is one of the reasons patients are recommended for presurgical fMRI,^28^ better identification of predictors of atypical language lateralization should improve patient selection protocols. In the LH group, the number of clinical predictors may serve as a good indicator of language lateralization at an individual level, with a greater number of predictors leading to increased confidence that the patient may be atypically lateralized. This cumulative effect may be due to the interaction between such predictors. For example, early LH acquired insults in isolation are associated with reorganization, but this association appears to be strengthened in cases where the insult involves the frontal lobe, and perhaps even more so if it is also accompanied by the reorganization of handedness functions.

### 4.6 Limitations

There was a high proportion of unexplained variance in our models. It is likely that the heterogeneity in task methodology between samples also contributed to the unexplained variance in our final models, as language tasks can be lateralizing to a different degree, and this interacts with the ROI used.^31^ This sample primarily included laterality indices calculated using a frontal ROI (89%); therefore, our findings likely reflect patterns and predictors of frontal language lateralization only. Given higher rates of crossed dominance in individuals with epilepsy,^1^ predictors of atypical language lateralization may vary based on whether frontal or temporal ROI were used for LI calculation. Indeed, only left/ambidextrous handedness and a history of stroke were also significant predictors of atypical temporal lobe language lateralization in the LH epilepsy group. In addition, there were no predictors of temporal language lateralization in the RH epilepsy group, and our overall models explained less variance in LI than the models predicting predominantly frontal language lateralization. This is despite atypical temporal language lateralization being more common in our sample.

## 5 CONCLUSION

Overall, our findings were largely consistent with theories of language plasticity proposing greater reorganization after early LH insult. Early LH acquired injuries were robust predictors of atypical language lateralization, particularly when combined with a frontal lobe location and atypical handedness. Other mechanisms beyond early plasticity may underlie atypical language lateralization in the wider sample, including increasing bilaterality with a longer duration of epilepsy. This suggests the presence of different mechanisms underlying atypical language lateralization in epilepsy: one associated with early developmental plasticity and another with the effect of chronic seizures. These findings predominantly reflect patterns of frontal lobe language lateralization, with only some replicated in the temporal lobe. Our cumulative predictor model provides a practical way to identify those who are more likely to demonstrate atypical language lateralization in the frontal lobe. However, high rates of atypical language lateralization in RH epilepsy sample suggests that presurgical fMRI should be considered for all surgical candidates when surgery would involve language or memory critical cortex.

## AUTHOR CONTRIBUTIONS

F.P., L.C., J.M., F.L., and T.B. contributed to the conception and design of this study. F.P., M.H.E., J.M., L.N.S., M.M.B., W.D.G., and T.B. contributed to the acquisition and analysis of data. F.P., M.H.E., F.L., and T.B. contributed to drafting a significant portion of the manuscript or figures.

## CONFLICT OF INTEREST STATEMENT

The authors have no conflicts of interest to declare. We confirm that we have read the Journal's position on issues involved in ethical publication and affirm that this report is consistent with those guidelines.

## Supporting information


Data S1.

## Data Availability

The data that support the findings of this study are available from the corresponding author upon reasonable request. For the small proportion of these data shared by the original study authors, requests will be forwarded onto the author of the original study.
