# Supplementary material for: Predictors of atypical language lateralization in focal epilepsy: A mega‐analysis of fMRI evidence
Source: Epilepsia. 2025 May 5;66(8):2842–52. doi: 10.1111/epi.18422 (PMC12371624; doi:10.1111/epi.18422)
Supplement: Supplementary file 1 — Data S1. [file EPI-66-2842-s001.docx]

**Supplementary materials**

The data extracted for each included study are summarized in Supplementary Table 1.

| *Supplementary Table 1. Characteristics of studies included in meta-analysis* | | | | |
| --- | --- | --- | --- | --- |
| **Study** | **Extraction** | **Participant characteristics** | **fMRI language task(s) and baseline condition** | **LI calculation method (voxel count vs magnitude, threshold)** |
| Adcock et al. (2003) | Individual participant data from article | N=19; left & right; TLE; mostly HS & tumors; adult sample with one pediatric case (age range: 15-54y); all right-handed as determined using EHI | Phonemic fluency and a visual fixation baseline | Frontal ROI. Magnitude of activation in ROI |
| Appel et al. (2012) | Individual participant data from article | N=33; left & right; TLE; mostly HS or MRI negative; adult sample (age range: 18-55y); handedness determined by clinical evaluation for 20 and with EHI for 13 | Auditory description decision and a  reverse speech baseline | Frontal ROI. LI-toolbox: Voxel count/value, mean LIs determined across different thresholds using bootstrapping methods in ROIs |
| Arora et al. (2009) | Individual participant data from article | N=5; left & right; mixed epilepsy locations; etiology not reported; adult and pediatric (age range: 12-51y); handedness determination method not-specified | Semantic and phonemic fluency*, visual sentence comprehension, auditory sentence comprehension and a line orientation judgement baseline | Whole brain ROI. Voxel count, threshold of t=2 across ROI (chosen out of a range of thresholds as it demonstrated the greatest stability) |
| Audrain et al. (2018) | Individual participant data from author | N=11; left only; TLE; etiology not specified; adult sample (age range: 23-58y); handedness determination method not-specified | Conjunction of 4 language tasks (covert verb generation, sentence comprehension, category fluency, naming to description) and a visual fixation baseline | Frontal ROI. LI-toolbox: Voxel count/value, mean LIs determined across different thresholds using bootstrapping methods in ROIs |
| Benjamin et al. (2017) | Individual participant data from article | N=14; left & right; mostly TLE, 1 frontal and 1 fronto-temporal; mixed etiology; adult sample with one pediatric case (age range: 16-56y); handedness determined by clinical evaluation | Conjunction of 3 lexico-semantic tasks (object naming, word reading, naming to description) and a rest baseline | Frontal + Temporal ROI. Voxel count, fixed threshold with a joint probability of p < 0.001 across ROI |
| Brazdil et al. (2005) | Individual participant data from article | N=13; left only; TLE; all HS; adult sample (age range: 19-53y); all right-handed as determined by self-report | Phonemic fluency and a rest baseline | Frontal ROI. Voxel count in ROI, threshold unreported |
| Carpentier et al. (2001) | Individual participant data from article | N=10; left only: mostly TLE, also frontal and temporoparietal; mixed etiology; adult sample (age range: 24-51y); handedness determination method not-specified | Conjunction of tasks (syntactic and semantic judgements on auditorily and visually presentenced sentences) and tone or line baselines | Frontal + temporal ROI. Voxel count, threshold of t>1.5 in ROI |
| Everts et al. (2010) | Individual participant data from article | N=40; left & right; frontal, temporal and other; mixed etiology; pediatric sample (age range: 7-17y); handedness determination method not-specified | Phonemic fluency and a rest baseline | Frontal ROI. LI-toolbox: Voxel count/value, mean LIs determined across different thresholds using bootstrapping methods in ROIs |
| Gross et al. (2022) | Individual participant data from author | N=77; left TLE; mixed etiologies; adult sample (age range: 18-68y); handedness determination method not-specified | Semantic decision and a tone decision baseline | Frontal + temporal ROI. Voxel count/value, mean LIs determined across different thresholds using bootstrapping methods in ROIs |
| Herfurth et al. (2022) | Individual participant data from article | N=8; left only; mostly temporal; mostly HS; adult sample (age range: 20-57y); handedness determined by EHI | Verb generation and a covert reading of nonsense syllable baseline | Frontal ROI. Sum of threshold surviving t-values per left  and right ROI |
| Koc et al. (2020) | Individual participant data from article | N=22; left & right; mostly TLE; mostly HS; adult sample (age range: 18-49y); all right-handed as determined by the EHI | Verb generation and a rest baseline | Frontal ROI. Count of voxels activated above p<0.05 in ROI |
| Kokkinos and Seimenis (2024) | Individual participant data from author | N=28; left & right; TLE; some HS; adult and pediatric sample (age range: 11-52y); handedness determined by EHI | Sentence generation* and reading comprehension^†^ and visual exploration baselines. Listening comprehension and a reverse speech baseline | Frontal ROI. Count of voxels activated in the BOLD maximum (area of highest statistical change with effect) in ROIs. |
| Norrelgen et al. (2015) | Individual participant data from article | N=11; left & right; TLE and some frontal and multifocal; mixed etiology; pediatric sample with two adults (age range: 8-18y); handedness determined by clinical evaluation or EHI | Verb generation* and a word repetition baseline. Story listening and a reverse speech baseline | Frontal ROI. Count of voxels activated above p<0.001 in ROIs |
| Sabbah et al. (2003) | Individual participant data from article | N=20; left & right: mostly TLE; mixed etiology; adult and pediatric sample (age range: 9-48y); handedness determination method not-specified | Semantic fluency and a rest baseline | Whole brain ROI. Count of voxels activated above p<0.0001 in ROI |
| Stasenko et al. (2022) | Individual participant data from article | N=9; left & right: TLE; mostly HS; adult sample (age range: 22-49y); handedness determination method not-specified | Semantic judgement and presentation of alphabet-like stimuli as a baseline | Frontal + temporal ROI. Count of voxels activated above p<0.01 in ROI |
| Szaflarski et al. (2008) | Individual participant data from article | N=26; left & right; mostly TLE; mostly HS; adult sample with one child (age range: 17-53y); handedness determined by EHI | Verb generation* and a finger tapping baseline. Semantic decision and a tone decision baseline | Frontal + temporal ROI. Count of voxels with z-scores ≥ 2.58 in ROI |
| Thivard et al. (2005) | Individual participant data from article | N=34; left & right; TLE: mostly HS; adult sample (age range: 18-55y); all right-handed as determined using the Harrel test of lateral dominance | Semantic fluency* and a rest baseline. Story listening and a reverse speech baseline | Frontal ROI. Count of voxels activated above p<0.05 (FWE) in ROIs |
| Tivarus et al. (2012) | Individual participant data from article | N=17; left only; mostly TLE; mostly HS; adult sample (age range: 39-69y); handedness determined by EHI | Conjunction of four language tasks (verb generation and a visual fixation baseline, semantic decision and a tone decision baseline, definition naming and a synthetic sound judgement baseline, passive sentence reading and a presentation of a sentence made up of alphabet-like stimuli as a baseline) | Frontal + temporal ROI. Count of voxels with z-scores ≥ 2.3 in ROI |
| Trimmel et al. (2019) | Individual participant data from author | N=45; left & right; TLE; etiology not specified; adult sample (age range: 19-58y); handedness determined by EHI | Phonemic fluency* and auditory naming^†^ and a visual fixation baseline | Frontal ROI. Count of voxels activated above p<0.05 (FWE) in ROIs |
| Voets et al. (2006) | Individual participant data from article | N=12; left only; TLE; mostly HS; mostly adult sample (age range: 15-53y); all right-handed, handedness determination method not-specified | Phonemic fluency and visual fixation baseline | Frontal ROI. Activation change in voxels with maximum activation ROI |
| Wilke et al. (2011) | Individual participant data from author | N=22; left & right; FLE and TLE; mixed etiology; pediatric sample with 2 adults (age range: 5-18y); handedness determined by EHI | Expressive letter task* and abstract image judgement baseline and receptive beep-stories task and tone listening baseline | Frontal ROI. LI toolbox: Voxel count/value, mean LIs determined across different thresholds using bootstrapping methods in ROIs |
| Yuan et al. (2006) | Individual participant data from article | N=13; left & right; mixed location and etiology, many MRI negative; pediatric sample with 2 adults (age range: 8-19y); handedness determined by EHI | Verb generation and a finger tapping baseline | Frontal ROI. Count of voxels activated above threshold calculated from the mean value of the t-statistics for all voxels within ROI |
| CNH sample | Additional sample | N=166; Left & right; mixed location and etiology including MRI negative; mostly pediatric sample with 17 adults (age range: 5-23y); handedness determined by clinical evaluation | Auditory description decision and reverse speech/tone detection baseline | Frontal ROI. LI-toolbox: Voxel count/value, mean LIs determined across different thresholds using bootstrapping methods in ROIs |
| GOSH sample | Additional sample | N=259; left & right; mixed location and etiology; pediatric sample with 1 adult (age range: 4-19y); handedness determined by clinical evaluation | Verb generation and white noise baseline | Frontal ROI. LI-toolbox: Voxel count/value, mean LIs determined across different thresholds using bootstrapping methods in ROI |

* Indicates the language task used if multiple were reported. ^†^ Indicates the language task used for the temporal LI if different from the task used for the frontal LI.

fMRI = functional magnetic resonance imaging; FDR = false discovery rate; FWE = family wise error; CNH = Children’s National Hospital; EHI = Edinburgh Handedness Inventory; GOSH = Great Ormond Street Hospital; HS = hippocampal sclerosis; LI = laterality index; ROI = region of interest; TLE = temporal lobe epilepsy.

**Interaction between side of epilepsy and all other variables**

*Supplementary Table 2: Fixed interaction effects between side of epilepsy and all other variables on language lateralization*

| **Variable** | **β** | **p** | **corrected p** |
| --- | --- | --- | --- |
| Age at seizure onset * LH epilepsy | -0.00 | .393 | .499 |
| Duration * LH epilepsy | -0.01 | .006** | .026* |
| Handedness * LH epilepsy | -0.12 | .297 | .480 |
| Frontal involvement * LH epilepsy | -0.29 | .011* | .030* |
| Temporal involvement * LH epilepsy | 0.04 | .687 | .687 |
| HS diagnosis * LH epilepsy | -0.11 | .300 | .480 |
| Stroke * LH epilepsy | -0.29 | .437 | .499 |
| Precipitating injury * LH epilepsy | -0.44 | <.001*** | .003** |

*p<0.05, **p<.01, ***p<.001

**Variables associated with handedness in LH and RH epilepsy**

There was an interaction between side of epilepsy and frontal involvement (β=1.84, p=.006) on handedness. This demonstrates that frontal lobe involvement was a predictor of left/ambidextrous handedness in the LH group, and this was significantly different from the influence of this variable on handedness in the RH group. See Supplementary Table 4 for all main and interaction effects.

*Supplementary Table 3: Fixed interaction effects between side of epilepsy and all other variables on left/ambidextrous handedness*

| **Variable** | **β** | **p** | **corrected p** |
| --- | --- | --- | --- |
| Left hemisphere epilepsy | 0.39 | .053 | .142 |
| Age at seizure onset * left hemisphere epilepsy | -0.04 | .117 | .234 |
| Duration of epilepsy * left hemisphere epilepsy | 0.02 | .283 | .453 |
| Frontal involvement * left hemisphere epilepsy | 1.84 | <.001*** | .006** |
| Temporal involvement * left hemisphere epilepsy | -0.12 | .798 | .798 |
| HS diagnosis * left hemisphere epilepsy | 0.15 | .749 | .798 |
| Stroke * left hemisphere epilepsy | 16.94 | .787 | .798 |
| Precipitating injury * left hemisphere epilepsy | 1.91 | .016* | .066 |

*p<0.05, **p<.01, ***p<.001

**Predictors of language lateralization using temporal ROIs**

In the LH epilepsy sample, 50% of individuals had atypical language lateralization in the temporal lobe (32% right lateralized, 18% bilateral) and the median LI was 0.21 (IQR=-0.07, 0.75). In the RH epilepsy sample, 32% of individuals had atypical language lateralization in the temporal lobe (21% right lateralized, 11% bilateral) and the median LI was 0.56 (IQR=0.32, 0.79). In the combined LH and RH sample, LH epilepsy was a significant predictor of greater atypical language lateralization (β=-0.22, p<.001).

For the LH multilevel model, the fixed effects explained 6% of the variance in LI, and the random effects explained an additional 12% (AIC=724). There was very strong evidence (BF>1000) that this model was a better fit for the data than one with random effects only (AIC=699), but the difference was not statistically significant (p<.001). Multicollinearity was low (VIF below 2). For the RH multilevel model, the fixed effects explained 7% of the variance in LI, and the random effects explained an additional 11% (AIC=393). There was strong evidence (BF>1000) that this model was a poorer fit for the data than one with random effects only (AIC=362), and there was no significant difference between the models (p>.05). Multicollinearity was low (VIF below 2). The estimates of the fixed effects for both the LH and RH epilepsy models are displayed in Supplementary Table 4.

*Supplementary Table 4. Estimates of fixed effects for LH and RH epilepsy sample multilevel models.*

| **Model** | **Fixed effects** | **Estimate** | **t** | **P value** |
| --- | --- | --- | --- | --- |
| **LH epilepsy** |  |  |  |  |
|  | Age at seizure onset | 0.00 | 0.49 | .623 |
|  | Duration of epilepsy | -0.01 | -1.62 | .107 |
|  | Left/ambidextrous handedness | -0.20 | -2.16 | .032* |
|  | Frontal lobe involvement | 0.11 | 1.17 | .242 |
|  | Temporal lobe involvement | -0.12 | -1.39 | .166 |
|  | HS diagnosis | 0.07 | 0.64 | .523 |
|  | Stroke | -0.35 | -2.13 | .034* |
|  | Other precipitating injury | -0.07 | -0.59 | .557 |
| **RH epilepsy** |  |  |  |  |
|  | Age at seizure onset | 0.00 | 0.43 | .669 |
|  | Duration of epilepsy | -0.00 | 0.25 | .802 |
|  | Left/ambidextrous handedness | -0.20 | -1.42 | .159 |
|  | Frontal lobe involvement | 0.16 | 1.06 | .293 |
|  | Temporal lobe involvement | 0.11 | 0.77 | .440 |
|  | HS diagnosis | 0.06 | 0.42 | .677 |
|  | Stroke | -0.06 | -0.13 | .901 |
|  | Other precipitating injury | 0.32 | 1.87 | .064 |

*p<0.05, **p<.01, ***p<.001
